# Application of fluorescent cholangiography to complex biliary variants of the confluence of the cystic duct and the infraportal type of the left lateral bile duct during single‐incision laparoscopic cholecystectomy: A case report

**DOI:** 10.1111/ases.13404

**Published:** 2024-11-07

**Authors:** Shinji Nishino, Tsuyoshi Igami, Yukihiro Yokoyama, Takashi Mizuno, Junpei Yamaguchi, Shunsuke Onoe, Masaki Sunagawa, Nobuyuki Watanabe, Taisuke Baba, Shoji Kawakatsu, Tomoki Ebata

**Affiliations:** ^1^ Division of Surgical Oncology, Department of Surgery Nagoya University Graduate School of Medicine Nagoya Japan

**Keywords:** fluorescent cholangiography, infraportal type of the left lateral bile duct, single‐incision laparoscopic cholecystectomy

## Abstract

A 21‐year‐old man was diagnosed with segmental adenomyomatosis of the gallbladder based on ultrasonography and computed tomography images. Computed tomography with drip infusion cholangiography revealed that the cystic duct joined the infraportal type of the left lateral bile duct (IPLLBD), which runs caudal to the umbilical portion, and that the left medial bile duct joined the right hepatic duct without forming the left hepatic duct. We planned a single‐incision laparoscopic cholecystectomy with fluorescent cholangiography. The fluorescent cholangiography visualized the anatomic variant of the biliary system, and the cystic duct was divided safely. Fluorescent cholangiography is a suitable procedure to depict complex biliary anatomic variations in this patient. IPLLBD without the formation of the left hepatic duct is potentially hazardous during cholecystectomy.

## 1 INTRODUCTION

Since fluorescent cholangiography (FC) was developed, it has been recognized as a suitable procedure to identify the biliary anatomy, including the accessory hepatic duct and the right‐sided round ligament, during laparoscopic cholecystectomy (LC) and/or single‐incision laparoscopic cholecystectomy (SILC).^1^, ^2^ Currently, FC is widely accepted as a suitable procedure to avoid biliary injury during LC and train a young surgeon on LC.^3^, ^4^, ^5^ Similarly, we proactively utilized FC during SILC and previously reported clinical values of FC for patients with the infraportal type of the right posterior bile duct, the right‐sided umbilical portion, and/or the cystohepatic duct.^6^, ^7^, ^8^ For anatomical variation of the left hepatic duct (LHD), only two reports described a rare anatomic variation in which the left lateral duct or its segmental duct runs caudally to the umbilical portion of the left portal vein, termed infraportal type.^9^, ^10^

Herein, we describe successful SILC in patients with rare biliary anatomical variation in which the cystic duct (CD) joins the infraportal type of the left lateral bile duct (IPLLBD) via FC.

## 2 CASE PRESENTATION

**FIGURE 1:**
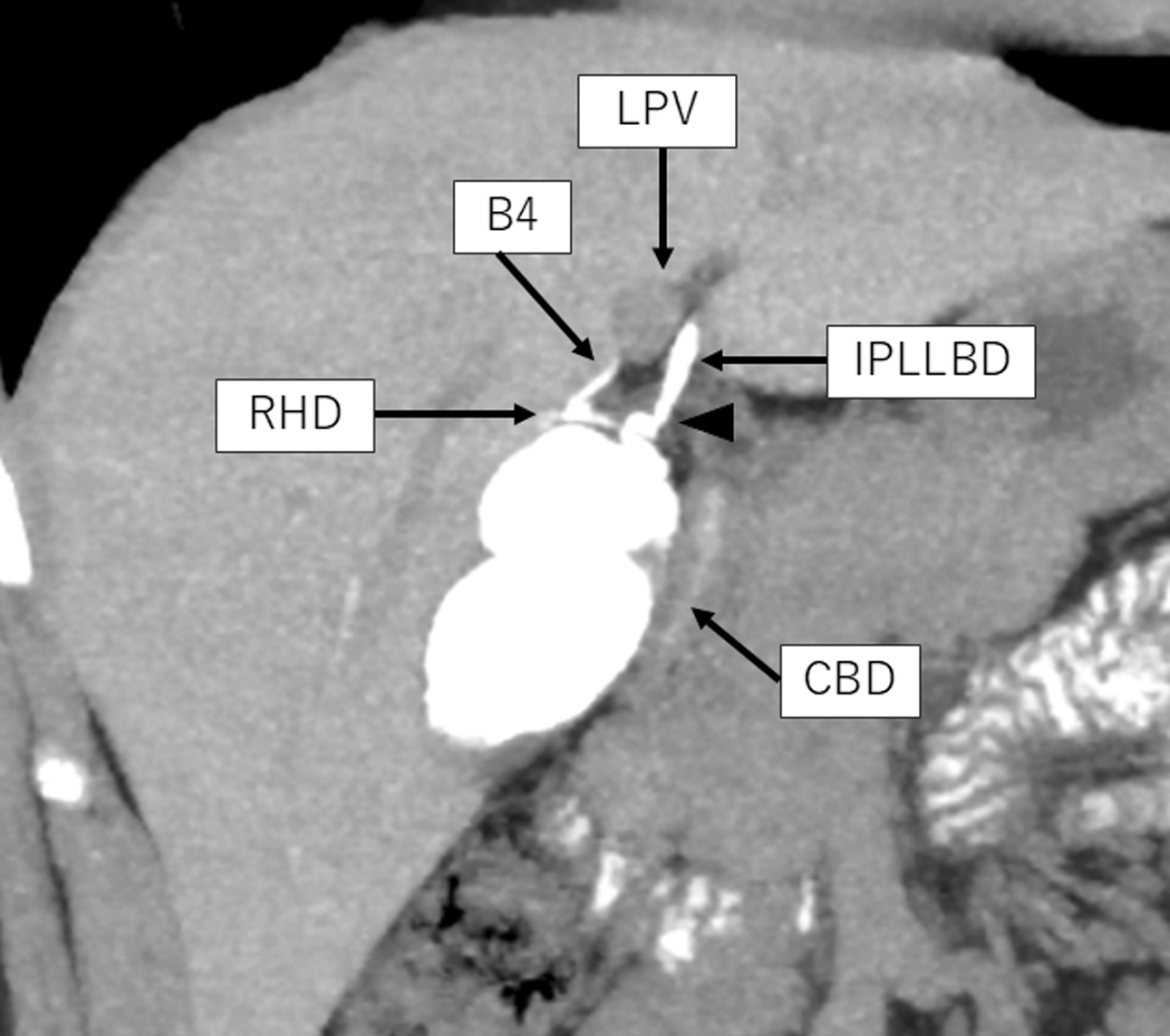
Computed tomography with drip infusion cholangiography revealed that the left lateral bile duct ran caudal to the umbilical portion, and this bile duct was defined as the infraportal type of the left lateral bile duct (IPLLBD). The left medial bile duct (B4) joins the right hepatic duct (RHD). The black arrowhead represents the confluence of the cystic duct and the IPLLBD. CBD, common bile duct; LPV, left portal vein.

A 21‐year‐old man presented with upper abdominal discomfort and was referred from a local clinic to our hospital. He was diagnosed with segmental adenomyomatosis of the gallbladder based on ultrasonography and multi‐detector raw computed tomography (MDCT) images (Figure S1). The three‐dimensional arteriography reconstructed from MDCT images revealed that the cystic artery came from the middle hepatic artery, and the three‐dimensional portography reconstructed from MDCT images revealed no findings of the right‐sided umbilical portion (Figure S2). Computed tomography with drip infusion cholangiography (DIC‐CT) revealed a complicated biliary anatomy with three variations (Figure 1). First, the left lateral bile duct runs caudally to the umbilical portion of the left portal vein, indicating IPLLBD. Second, the CD is joined into the IPLLBD. The left medial bile duct (B4) directly joins the right hepatic duct (RHD), not the LHD. The schema of the vascular and biliary anatomy is shown in Figure S3. Because of the patient's strong desire for minimally invasive surgery, we planned the SILC with reference to the FC.

In accordance with our previous reports,^1^, ^2^, ^3^, ^4^ SILC with FC was performed. After a single 2.5‐cm‐long vertical incision was made across the umbilicus, a SILS port (Covidien, Mansfield, MA, USA) was placed through it. As a fluorescent source, 1 mL of indocyanine green (2.5 mg/mL of Diagnogreen®; Daiichi Sankyo Co., Tokyo, Japan) was intravenously injected after endotracheal intubation of the patient in the operating room. A D‐light P‐light source unit (Karl Storz Endoskope, Tuttlingen, Germany) with the ability to alternate between xenon and infrared light via a foot pedal served as a laparoscopic fluorescence imaging system via a 5‐mm port inside the SILS port. When anatomical visualization of the biliary structures was necessary, FC was repeated.

**FIGURE 2:**
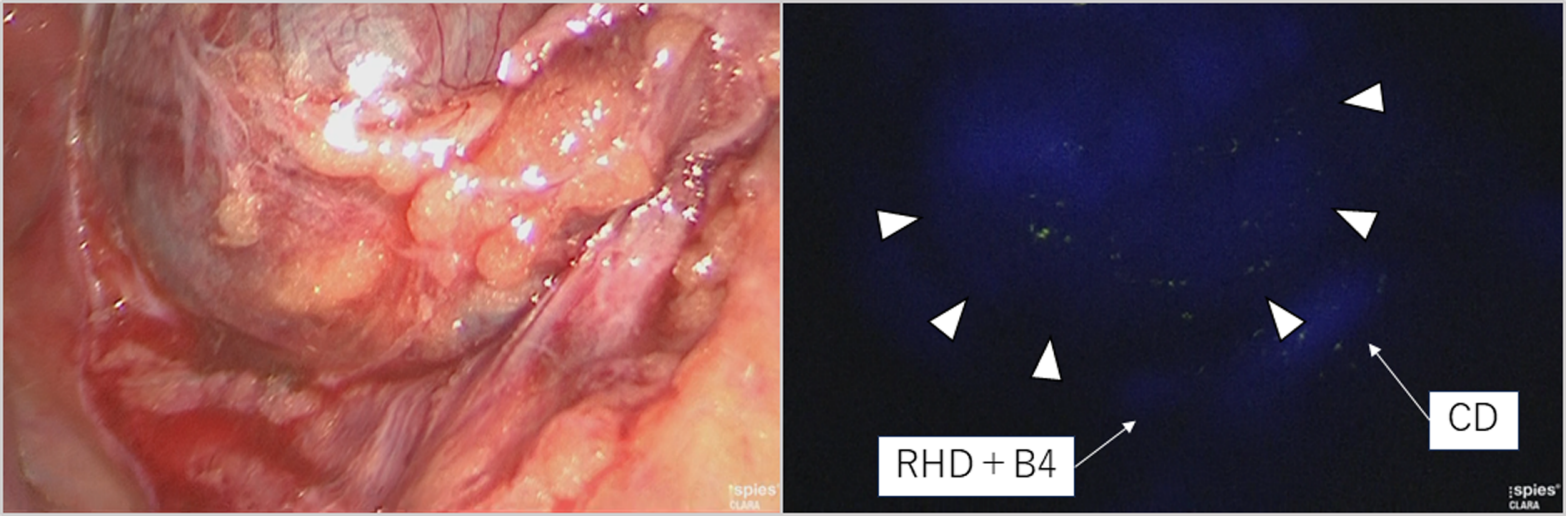
Intraoperative findings during dissection caudal to the neck of the gallbladder. The normal laparoscopic view (the left‐sided view) revealed that the biliary structures were unclear during dissection caudal to the neck of the gallbladder. Under fluorescent cholangiography (right‐sided view), the neck of the gallbladder (white arrowheads) overlaid the confluent branch of the right hepatic duct (RHD), and the left medial bile duct (RHD + B4) and the cystic duct (CD) were well visualized.

**FIGURE 3:**
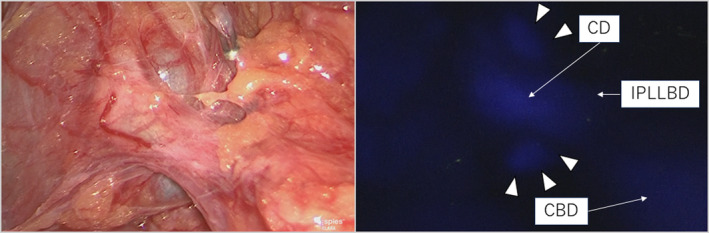
Intraoperative findings confirming the confluence of the cystic duct. The normal laparoscopic view (the left‐sided view) revealed that the confluence of the cystic duct was unclear. Under fluorescent cholangiography (right‐sided view), the cystic duct (CD) runs caudal to the neck of the gallbladder (white arrowheads) and ventral to the common bile duct (CBD) and joins the infraportal type of the left lateral bile duct (IPLLBD).

**FIGURE 4:**
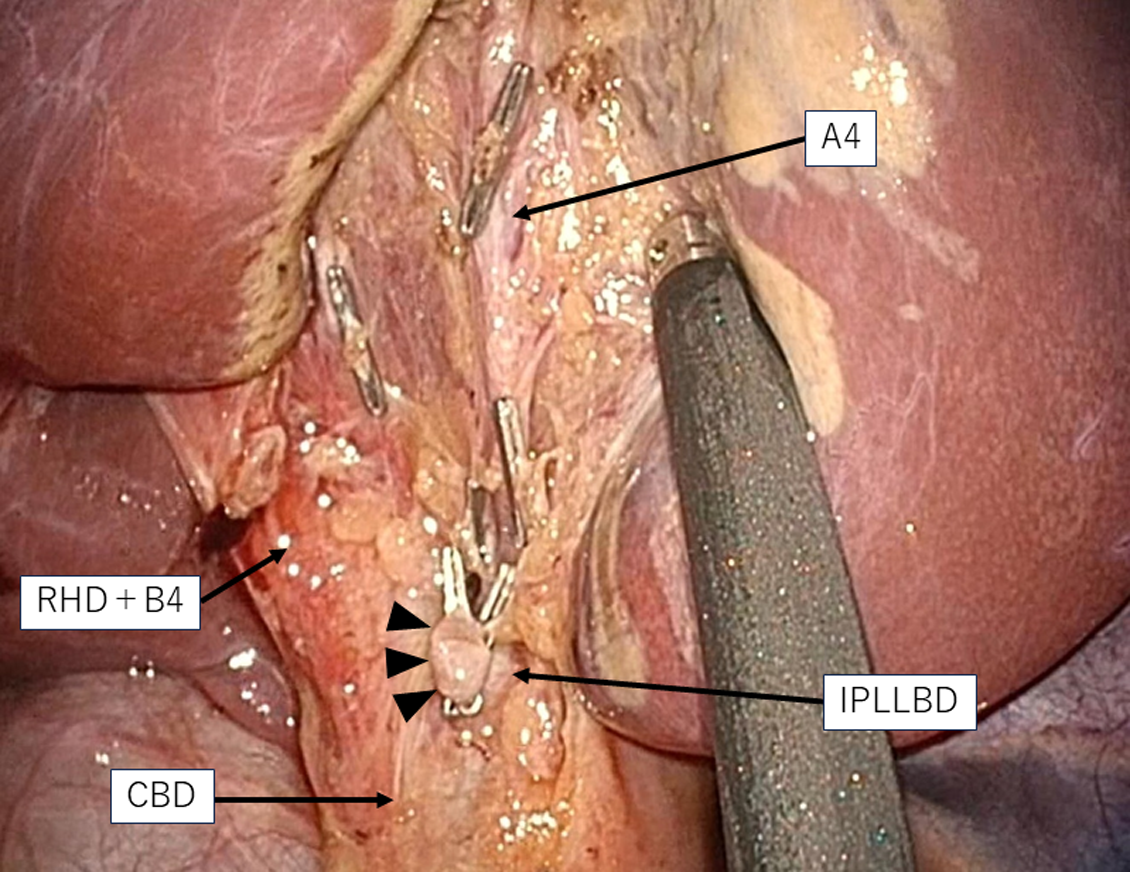
Intraoperative findings after resection of the gallbladder. Under normal laparoscopic view, the stump of the cystic duct (black arrowheads) joins the intraportal type of the left lateral bile duct (IPLLBD), the common bile duct (CBD) runs dorsal to the stump of the cystic duct (black arrowheads), and the confluent branch of the right hepatic duct (RHD) and the left medial bile duct (RHD + B4) runs cranially to the stump of the cystic duct (black arrowhead). The middle hepatic artery (A4) was exposed on the gallbladder bed. Those important anatomical structures had no intraoperative injuries.

The gallbladder located between segments 4 and 5 and its wall had no inflammation (Figure S4); however, the clinical view of safety (CVS) was quite difficult to achieve because the neck of the gallbladder overlaid the RHD and B4 (Figure 2). Dissection around the neck of the gallbladder was carefully performed with repeated utilization of FC. A normal laparoscopic view failed to show the confluence of the CD, whereas the FC clearly showed the confluence of the CD and IPLLBD (Figure 3); therefore, the CD was divided safely. Intraoperative findings after removal revealed no injuries to the anatomical structures (Figure 4). The operative time and amount of intraoperative blood loss were 232 min and 3 mL, respectively. A histological diagnosis of adenomyomatosis of the gallbladder was confirmed. He was discharged 3 days after surgery without any postoperative complications.

## 3 DISCUSSION

The clinical utility of FC during LC and/or SILC has been accepted because FC‐guided LC facilitates dissection of Calot's triangle easily through clear visualization of the biliary system, including the accessory hepatic duct and the right‐sided round ligament.^1^, ^2^ Recently, FC‐guided LC is recognized as a valuable procedure to avoid biliary injury and train a young surgeon on LC.^3^, ^4^, ^5^ Similarly, we reported the clinical value of FC for the intraoperative identification of anatomical variations of the bile duct, that is, the infraportal type of the right posterior bile duct, the right‐sided umbilical portion, and/or the cystohepatic duct.^6^, ^7^, ^8^ Even when the CVS was unsafe to achieve, gradual dissection around the neck of the gallbladder under repeated guidance with FC enabled the safe completion of SILC. Accordingly, in such a biliary variation, FC is a suitable procedure to avoid biliary injury.

The left lateral bile duct usually runs cranially to the umbilical portion of the left portal vein, and the frequency of the infraportal type B3 varies from 3.3% to 8.3%.^9^, ^10^ In our series of 569 patients whose biliary anatomy was evaluated before LC, only one patient had IPLLBD (0.18%); therefore, IPLLBD was a rare biliary anatomical variation. Additionally, in this patient, the LHD was not formed because B4 joined the RHD. In such situations, right hemihepatectomy potentially carries the risk of unintended injury to B4.

In conclusion, FC can be recognized as an easy and suitable procedure for patients with a confluence of CD and IPLLBD to avoid biliary injury during SILC.

## CONFLICT OF INTEREST STATEMENT

The authors have no relevant financial disclosures.

## PATIENT CONSENT STATEMENT

We obtained informed consent from the patient.

## Supporting information


**Figure S1.** Multi‐detector raw computed tomography with enhanced contrast. The gallbladder (GB) is located between segments 4 and 5, and its wall is thick. The round ligament (RL) is located normally. P3, ventral left lateral portal vein; P4, medial portal vein.

**Figure S2.** The three‐dimensional angiography and portography reconstructed from multi‐detector raw computed tomography. The three‐dimensional angiography revealed that the cystic artery (CA) came from the middle hepatic artery (MHA). The three‐dimensional portography revealed no findings of the right‐sided round ligament. LHA, left hepatic artery; LPV, left portal vein; RAPV, right anterior portal vein; RHA, right hepatic artery; RPPV, right posterior portal vein.

**Figure S3.** Schema of the vascular and biliary anatomy. The cystic duct (CD) joins the infraportal type of the left lateral bile duct (IPLLBD). The left medial bile duct (B4) directly joins the right hepatic duct (RHD), not the left hepatic duct. The cystic artery (CA) comes from the middle hepatic artery (MHA). LHA, left hepatic artery; LPV, left portal vein; RHA, right hepatic artery; RPV, right portal vein.

**Figure S4.** Intraoperative findings before dissection of Calot's triangle. The gallbladder is located between segments 4 (S4) and 5 (S5), and its wall had no inflammation. RL, round ligament; RS, Rouviere's sulcus.

## Data Availability

The data that support the findings of this study are available from the corresponding author upon reasonable request.

## References

[1] Ishizawa T , Bandai Y , Ijichi M , Kaneko J , Hasegawa K , Kokudo N . Fluorescent cholangiography illuminating the biliary tree during laparoscopic cholecystectomy. Br J Surg. 2010;97:1369‐1377. 20623766 10.1002/bjs.7125

[2] Ishizawa T , Kaneko J , Inoue Y , et al. Application of fluorescent cholangiography to single‐incision laparoscopic cholecystectomy. Surg Endosc. 2011;25:2631‐2636. 21424202 10.1007/s00464-011-1616-2

[3] Dip F , Lo Menzo E , White KP , Rosenthal RJ . Does near‐infrared fluorescent cholangiography with indocyanine green reduce bile duct injuries and conversions to open surgery during laparoscopic or robotic cholecystectomy? A meta‐analysis. Surgery. 2021;169:859‐867. 33478756 10.1016/j.surg.2020.12.008

[4] Lim SH , Tan HTA , Shelat VG . Comparison of indocyanine green dye fluorescent cholangiography with intra‐operative cholangiography in laparoscopic cholecystectomy: a meta‐analysis. Surg Endosc. 2021;35:1511‐1520. 33398590 10.1007/s00464-020-08164-5

[5] Ortenzi M , Corallino D , Botteri E , et al. Safety of laparoscopic cholecystectomy performed by trainee surgeons with different cholangiographic techniques (SCOTCH): a prospective non‐randomized trial on the impact of fluorescent cholangiography during laparoscopic cholecystectomy performed by trainees. Surg Endosc. 2024;38:1045‐1058. 38135732 10.1007/s00464-023-10613-w

[6] Igami T , Asai Y , Minami T , et al. Clinical value of fluorescent cholangiography for the infraportal type of right posterior bile duct. Minim Invasive Ther Allied Technol. 2023;32:256‐263. 37288773 10.1080/13645706.2023.2217915

[7] Nojiri M , Igami T , Toyoda Y , et al. Application of fluorescent cholangiography during single‐incision laparoscopic cholecystectomy for cholecystitis with a right‐sided round ligament: preliminary experience. J Minim Access Surg. 2018;14:244‐246. 29226884 10.4103/jmas.JMAS_159_17 PMC6001308

[8] Asai Y , Igami T , Ebata T , et al. Application of fluorescent cholangiography during single‐incision laparoscopic cholecystectomy in the cystohepatic duct without preoperative diagnosis. ANZ J Surg. 2021;91:470‐472. 32681758 10.1111/ans.16162

[9] Okubo M , Nagino M , Kamiya J , et al. Surgical anatomy of the bile ducts at the hepatic hilum as applied to living donor liver transplantation. Ann Surg. 2004;239:82‐86. 14685104 10.1097/01.sla.0000102934.93029.89 PMC1356196

[10] Ozden I , Kamiya J , Nagino M , Uesaka K , Sano T , Nimura Y . Clinicoanatomical study on the infraportal bile ducts of segment 3. World J Surg. 2002;26:1441‐1445. 12297940 10.1007/s00268-002-6544-9

