# Supplementary figures and images for: Application of fluorescent cholangiography to complex biliary variants of the confluence of the cystic duct and the infraportal type of the left lateral bile duct during single‐incision laparoscopic cholecystectomy: A case report
Source: Asian J Endosc Surg. 2024 Nov 7;18(1):e13404. doi: 10.1111/ases.13404 (PMC12116224; doi:10.1111/ases.13404)

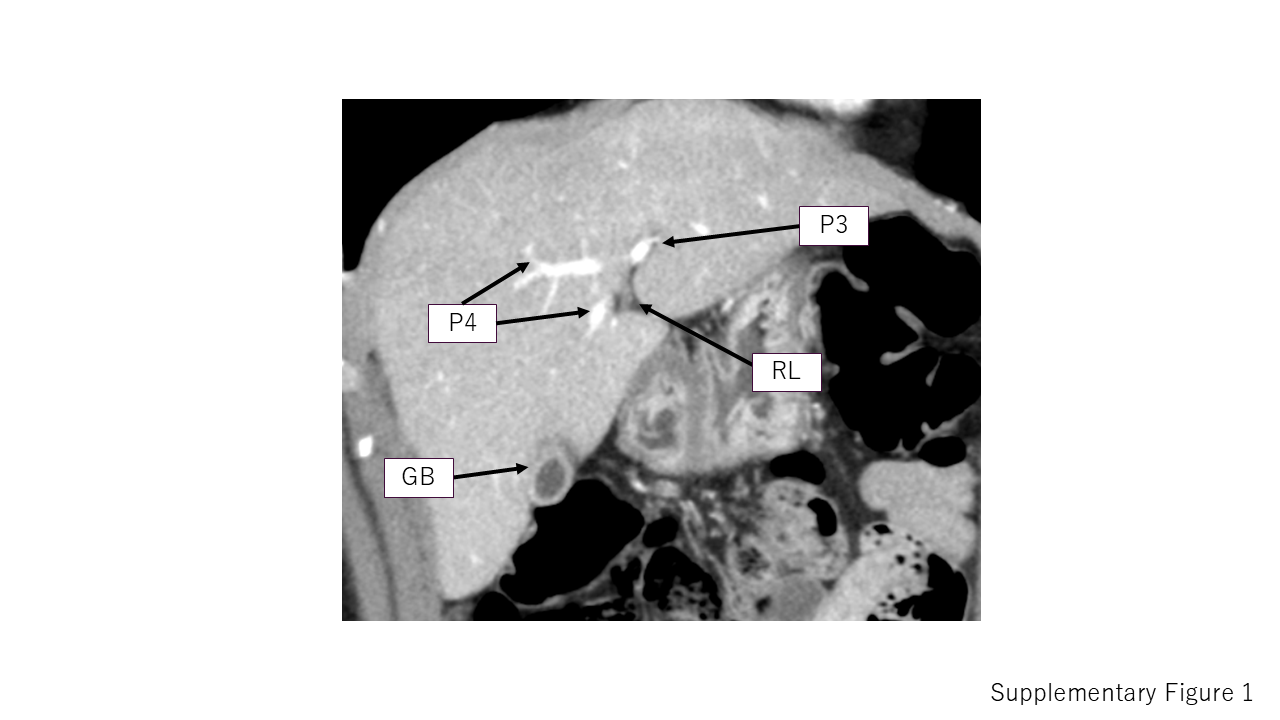

Supplement: Supplementary file 1 — Figure S1. Multi‐detector raw computed tomography with enhanced contrast. The gallbladder (GB) is located between segments 4 and 5, and its wall is thick. The round ligament (RL) is located normally. P3, ventral left lateral portal vein; P4, medial portal vein. [file ASES-18-e13404-s003.tif]

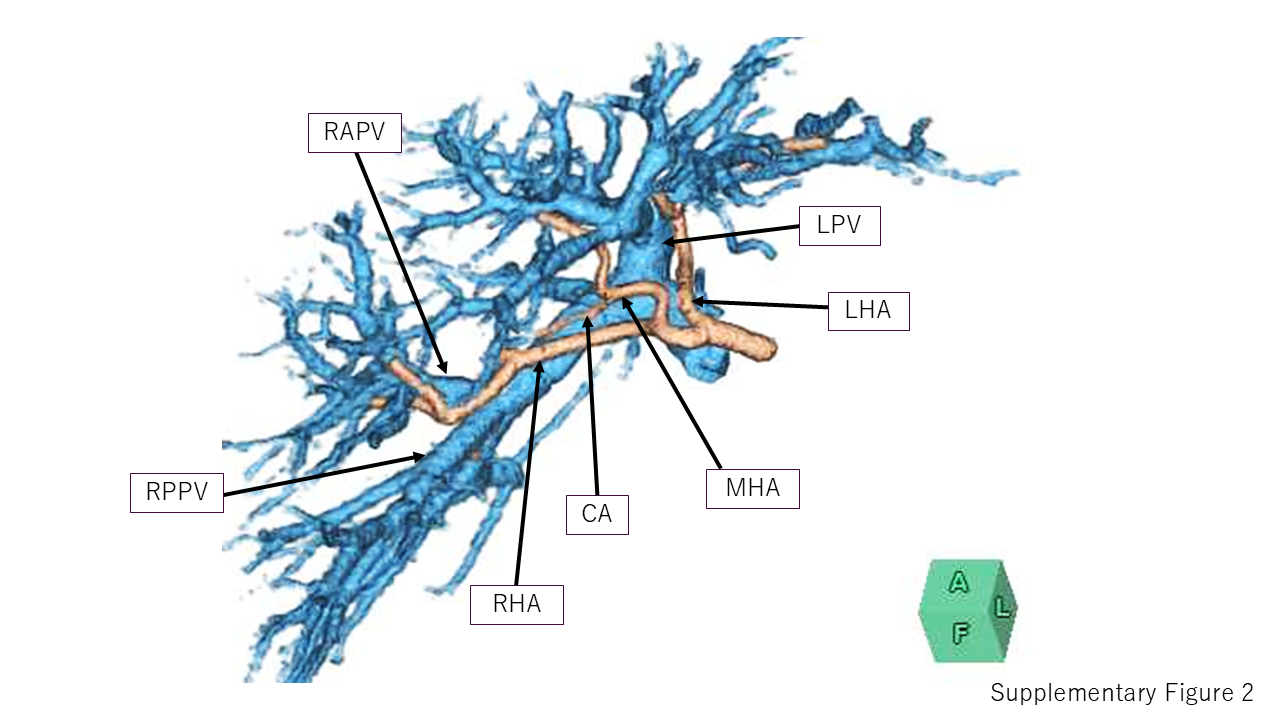

Supplement: Supplementary file 2 — Figure S2. The three‐dimensional angiography and portography reconstructed from multi‐detector raw computed tomography. The three‐dimensional angiography revealed that the cystic artery (CA) came from the middle hepatic artery (MHA). The three‐dimensional portography revealed no findings of the right‐sided round ligament. LHA, left hepatic artery; LPV, left portal vein; RAPV, right anterior portal vein; RHA, right hepatic artery; RPPV, right posterior portal vein. [file ASES-18-e13404-s001.tif]

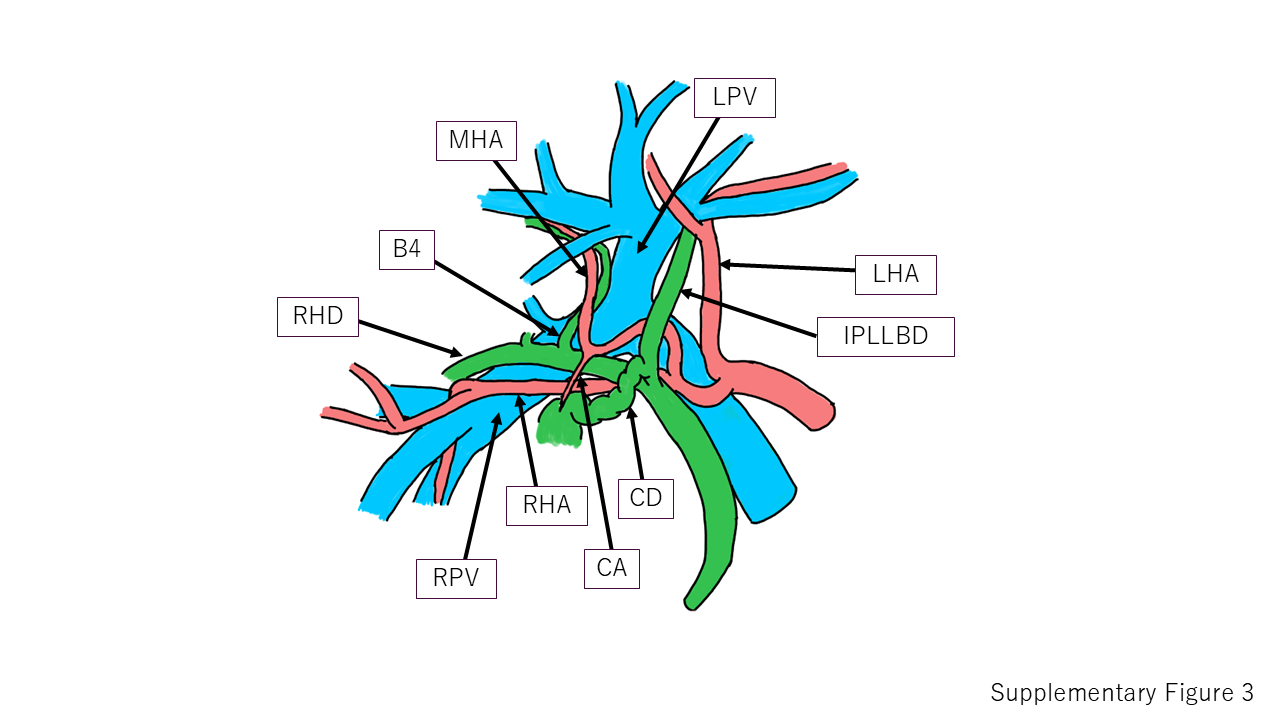

Supplement: Supplementary file 3 — Figure S3. Schema of the vascular and biliary anatomy. The cystic duct (CD) joins the infraportal type of the left lateral bile duct (IPLLBD). The left medial bile duct (B4) directly joins the right hepatic duct (RHD), not the left hepatic duct. The cystic artery (CA) comes from the middle hepatic artery (MHA). LHA, left hepatic artery; LPV, left portal vein; RHA, right hepatic artery; RPV, right portal vein. [file ASES-18-e13404-s002.tif]

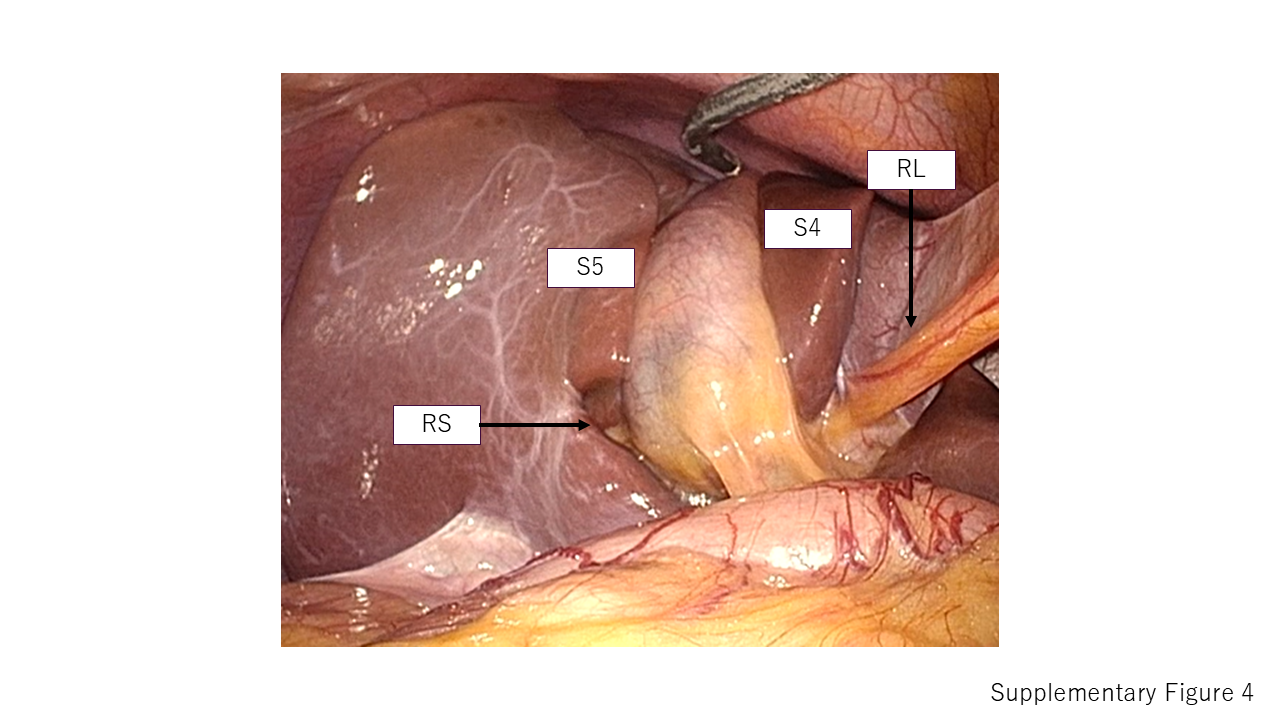

Supplement: Supplementary file 4 — Figure S4. Intraoperative findings before dissection of Calot's triangle. The gallbladder is located between segments 4 (S4) and 5 (S5), and its wall had no inflammation. RL, round ligament; RS, Rouviere's sulcus. [file ASES-18-e13404-s004.tif]
